# Kerala’s Aardram Mission and universal health coverage: integrated time series evidence on mortality reduction and assessment of financial protection and equity gaps

**DOI:** 10.3389/fpubh.2026.1882081

**Published:** 2026-08-05

**Authors:** Kolappatta Shahana, Balamurugan Janarthanan

**Affiliations:** Department of Social Sciences, School of Social Sciences and Languages, VIT University, Vellore, India

**Keywords:** Aardram Mission, financial protection barriers, health policy implementation, implementation gaps, Kerala, maternal infant mortality reduction, primary health care, universal health coverage

## Abstract

**Introduction:**

Universal health coverage (UHC) requires simultaneous progress across health outcomes, financial protection, and service delivery dimensions. Kerala has achieved favorable mortality indicators, yet implementation gaps and financing barriers persist despite policy reforms. The Aardram Mission (2017) represents a major primary care strengthening intervention aimed at advancing UHC, but its association with multiple UHC dimensions has not previously been examined in an integrated way.

**Objective:**

The study aimed to assess the association between the implementation of the Aardram Mission and changes in maternal and infant mortality, health insurance coverage, immunization rates, and out-of-pocket expenditure patterns and to identify implementation gaps and health equity disparities within Kerala’s health system..

**Methods:**

The study analyzed secondary annual data from 2010 to 2024, covering the pre-policy (2010–2016) and post-policy (2017–2024) periods. Interrupted time series (ITS) with segmented regression was used as the primary analytical method to examine changes in the trajectories of the maternal mortality ratio (MMR), infant mortality ratio (IMR), and immunization coverage associated with the implementation of the Aardram Mission in 2017. Descriptive pre–post comparisons, correlation analysis, and multivariable regression were conducted as supporting exploratory analyses. The robustness of the ITS findings was assessed through sensitivity analyses accounting for the COVID-19 pandemic and a structural break test..

**Results:**

Segmented regression indicated an acceleration in the declining trends of both mortality indicators following the 2017 policy implementation, after accounting for pre-existing trends. Between 2017 and 2024, the IMR declined by 43.8% and the MMR by 47.9% relative to pre-policy levels, corresponding to an estimated 2.46-fold increase in the rate of IMR decline. These findings are consistent with, but do not independently establish, a causal policy effect, given the observational single-group design and the absence of a comparator series. Insurance coverage expanded substantially over the same period. Implementation gaps persisted in relation to out-of-pocket expenditure, which remained high.

**Conclusion:**

The Aardram Mission’s implementation coincided with meaningful improvements in mortality trends, but these findings do not, on their own, establish that the policy fully resolved Kerala’s UHC dimensions. Financial protection remains incomplete, implementation barriers appear to constrain service quality, and equity gaps persist. Comprehensive UHC in Kerala likely requires complementary governance and financing reforms extending beyond primary care strengthening.

## 1 Introduction

Universal Health Coverage (UHC) has become a cornerstone of global health policy, reflecting the commitment that everyone should have access to quality health services without being pushed into financial hardship as a result of using them (1). The WHO’s definition encompasses three key dimensions: Access to quality essential health services, financial risk protection, and health equity across populations (2). These dimensions are not separate in practice. Access without financial protection exposes families to catastrophic health expenditure via a different route, while financial protection without service availability does not constitute meaningful coverage (3). UHC is embedded within Sustainable Development Goal (SDG) 3 and the broader equity agenda (4), and the evidence base has converged on primary health care (PHC) strengthening as one of the more cost-effective pathways toward achieving UHC (5).

Despite global commitments to UHC, considerable implementation gaps persist between expanding healthcare coverage and ensuring financial protection (6). Many countries have achieved high levels of insurance enrollment but still experience catastrophic health expenditure and unmet needs (7); out-of-pocket expenditure remains a major barrier to healthcare access in low- and middle-income countries (LMICs) (8). Why the gaps persist seems to depend on where you look; in sub-Saharan Africa, they have been attributed to resource constraints and conflicting sector priorities (9), while evidence elsewhere shows that out-of-pocket expenditure patterns vary considerably even under nominal UHC arrangements (10). This suggests that UHC is not just a financial mechanism but a multidimensional construct (11).

India has pursued UHC through multiple national interventions, although progress has varied considerably across states. The National Health Mission (NHM), 2005, has shown measurable associations with reductions in mortality (12). India’s approach to UHC remains fragmented, comprising parallel systems of public provision, private sector engagement, and health insurance schemes (13). Out-of-pocket expenditure remains a challenge among low-income households (14), while government spending remains low relative to needs (15). The Ayushman Bharat program (2018) marked a shift toward strengthening primary care and expanding insurance coverage (15), but state-level evidence on its impact on actual UHC outcomes remains limited (16).

Kerala represents an outlier within India’s health system, achieving health outcomes comparable to middle-income countries despite substantially lower levels of health expenditure (17). The state has demonstrated markedly lower maternal and infant mortality rates than the national average, positioning it close to several UHC benchmarks (2). Despite high service utilization and low mortality rates, concerns remain regarding the quality and equity of care, the sustainability of financial protection mechanisms, and the extent to which genuine financial protection has been achieved (16). It is still unclear whether Kerala’s favorable mortality trends reflect comprehensive UHC implementation or whether they largely predate and remain independent of recent health system reforms. This “Kerala paradox”—favorable population health outcomes despite persistent implementation gaps and equity challenges—warrants empirical examination to better understand UHC implementation within Indian states.

Kerala’s favourable health outcomes predate the Aardram Mission and are widely attributed to structural and social investments made over several decades. The “Kerala model of development” is characterized by sustained investments in female education, land reform, decentralized governance, and public provision of primary health care (PHC) since the Travancore–Cochin period (16). The state also underwent a demographic and epidemiological transition, characterized by high female literacy and earlier declines in fertility and mortality rates compared to most other Indian states. Thus, the Aardram Mission should be assessed as a reform implemented within an already established health system rather than as the primary cause of these achievements. This perspective informed the use of interrupted time series (ITS) analysis, which estimates changes in level and trend after the 2017 intervention while accounting for underlying pre-intervention trends.

In 2017, the Government of Kerala launched the Aardram Mission under the Nava Kerala Mission as a comprehensive health system reform, restructuring primary care delivery through the establishment of family health centers (FHCs), modernization of taluk and district hospitals, and expansion of referral and specialty services (16, 17). By transforming existing sub-health centers and primary health centers (PHCs) into comprehensive facilities offering preventive, promotive, curative, rehabilitative, and palliative care, the mission aligns with global PHC strengthening priorities and the Ayushman Bharat framework’s commitment to UHC (13, 18). Understanding the associations between the mission and health system outcomes, the plausible mechanisms of change, and persistent gaps is essential for informing future health system strengthening efforts in Kerala and comparable Indian states.

Despite Kerala’s prominence in India’s health discourse and the significance of the Aardram Mission, critical gaps remain in understanding its implementation (19). Existing studies have primarily focused on either describing the policy reform itself or examining specific health outcomes such as mortality indicators, without providing an integrated analysis of how the policy is associated with multiple dimensions of UHC simultaneously. The relationship between policy implementation and health system gaps, including inadequacies in service delivery quality, persistent financing barriers, and equity issues, remains poorly understood (20). This study addresses this gap through systematic empirical analysis of the associations between the Aardram Mission and multiple dimensions of UHC in Kerala. The study’s primary objective is to examine, using ITS analysis of pre-policy (2010–2016) and post-policy (2017–2024) trends, whether the trajectory of maternal and infant mortality changed following the policy’s implementation, beyond pre-existing secular trends. A secondary objective is to describe system-level associations between health system strengthening investments and mortality outcomes using multivariable regression and correlation analyses while explicitly treating these as correlational rather than causal evidence. Specifically, the study addresses four questions: (1) What changes in maternal, infant, and child health outcomes coincided with the Aardram Mission’s implementation? (2) Did the policy period show evidence of genuine financial protection or do out-of-pocket and catastrophic health expenditures persist? (3) What gaps in service readiness, immunization coverage, and health equity remain despite expanded primary care provision? and (4) What governance and implementation factors may explain the policy’s apparent achievements and limitations. In addressing these questions, the study provides evidence-based insight into the extent to which the Aardram Mission is associated with movement toward genuine UHC in Kerala, while remaining explicit about the limits of causal inference that a single-group observational design permits.

This study employs a dual theoretical framework combining the World Health Organization’s UHC services and financial protection lens with political economy perspectives on health system constraints (1, 20). The WHO framework conceptualizes UHC as requiring expansion across four interconnected dimensions—population, service, financial, and quality coverage (14)—moving beyond simple enrollment metrics to examine whether expanded coverage translates into genuine access and protection (2). Political economy perspectives emphasize that health system performance is shaped not by technical factors but by resource availability, governance capacity, equity considerations, and political–institutional environment (1, 2).

## 2 Methods

### 2.1 Study design

This study employed an ITS analysis with segmented regression as its primary analytical approach. ITS is a quasi-experimental design recommended for evaluating population-level policy interventions in settings where comparable population control is unavailable, as it constructs an internal counterfactual from the pre-intervention trend rather than relying on a between-group comparison (18). This design was selected specifically because the Aardram Mission constitutes a clearly demarcated, dated policy intervention (2017) implemented statewide, precluding a contemporaneous within-state control group; continuous annual observations spanning 2010–2024 (*N* = 15) permit estimation of a stable pre-policy trend against which post-policy deviations can be assessed. The segmented regression framework operationalizes a counterfactual by extrapolating the pre-policy trend (*β*₁), and any treatment-coinciding effect is quantified as the deviation of the observed post-policy trajectory from this expected pathway. All other analyses reported in this study — descriptive comparisons, correlation analysis, and multivariable regression — are explicitly positioned as supporting or exploratory analyses subordinate to the ITS findings (see Section 2.5), since they draw on the same underlying time series and are not statistically independent sources of evidence.

### 2.2 Policy context and study period

The Aardram Mission (2017) is a state-level primary health care reform centered on transforming PHCs into FHCs. The study period (2010–2024) was divided into a pre-policy phase (2010–2016; *n* = 7) and a post-policy phase (2017–2024; *n* = 8). This division ensured temporal homogeneity within each phase and provided sufficient observations for baseline trend estimation. The pre-2017 trend served as the counterfactual baseline. As the post-policy period overlaps with the COVID-19 pandemic (2020–2022), potential confounding was addressed through the sensitivity analyses reported in Section 2.5.1. Given the Aardram Mission’s multiple reform components, this analysis focused on indicators for which consistent annual administrative data were available throughout 2010–2024: FHC expansion, health financing, service utilization, preventive care, and mortality outcomes.

### 2.3 Data sources and data harmonization

A longitudinal dataset spanning 2010–2024 was constructed from secondary administrative sources, including Health at a Glance: Kerala reports, Kerala State Budget documents, and Sample Registration System (SRS) reports, all of which provide continuous annual observations suitable for ITS analysis without interpolation. The National Family Health Survey (NFHS) (rounds 4 and 5) and National Sample Survey (NSS) datasets, collected at irregular multi-year intervals, were unsuitable for direct incorporation into segmented regression (18) and were instead used for external validation: Administrative estimates were cross-checked against survey-based estimates for corresponding years (2015–2016 and 2019–2021) to confirm plausibility and detect any systematic divergence between data sources. No interpolation, extrapolation, or imputation was applied to the series used in statistical modeling. Where indicators were reported by more than one source, operational definitions were harmonized to a single consistent measurement approach prior to aggregation. For example, insurance coverage and outpatient visit figures from Health at a Glance: Kerala reports were cross-checked against NFHS estimates for definitional consistency (population base and reference period) before being incorporated into the annual series. All data were aggregated at the state level into a single annual time series and partitioned into pre- and post-policy phases for comparative analyses. To enhance transparency and reproducibility, a detailed description of all study variables—operational definitions, measurement units, original data sources, data availability by year, and analytical role—is provided in Supplementary Table S1 and summarized in Table 1.

**Table 1:** Study variables, roles, and sources.

Variable	Role	Unit	Source	Analytical purpose
Health budget	Predictor	₹ crores	Kerala State Budget documents	Financing input; ITS and regression predictor
Insurance coverage	Predictor (proxy)	% population	Health at a Glance: Kerala; validated against NFHS 4/5	Enrollment-based proxy for financial protection, not a direct measure of catastrophic expenditure
Outpatient visits	Predictor	Per 1,000 population	Health at a Glance: Kerala; validated against NFHS 4/5	System-level proxy for service delivery and utilization intensity
FHC expansion	Predictor	Number of facilities	Government administrative records	Infrastructure expansion indicator
Immunization coverage	Outcome	%	Health at a Glance: Kerala; validated against the NFHS	Prevention dimension of UHC
IMR	Outcome	Per 1,000 live births	SRS reports	Primary mortality outcome
MMR	Outcome	Per 100,000 live births	SRS reports	Primary mortality outcome

Indicator-specific sources were as follows: Health budget allocations (Kerala State Budget documents), insurance coverage and outpatient visits (Health at a Glance: Kerala reports, validated against NFHS 4/5), FHC expansion (government administrative records), immunization coverage (Health at a Glance: Kerala, validated against the NFHS), and the infant mortality ratio (IMR)/ maternal mortality ratio (MMR) (SRS reports).

### 2.4 Variables

Indicators were selected to reflect key dimensions of UHC and the theoretical mechanisms through which the Aardram Mission is hypothesized to be associated with health outcomes. Table 1 summarizes each variable’s analytical role, unit, source, and purpose.

Financing variables included health budget allocation and insurance coverage. Insurance coverage was used in this study strictly as a proxy indicator of financial protection, reflecting the expansion of financial risk pooling and enrollment mechanisms; it does not directly measure realized financial protection, which the WHO UHC framework defines more broadly to include catastrophic health expenditure and the out-of-pocket expenditure burden. Continuous annual time series data on catastrophic health expenditure were not available for the full 2010–2024 study period at the required frequency (such measures are derived from periodic NSS/NFHS survey rounds; Section 2.3) and, therefore, could not be incorporated into the ITS models. Consequently, insurance coverage should be interpreted throughout this study as an enrollment proxy rather than a direct outcome measure of financial protection.

Service delivery was analyzed using aggregated public health system outpatient visits per 1,000 population, capturing utilization across all public health facilities, including PHCs, FHCs, taluk hospitals, and district hospitals. This indicator serves as a system-level proxy for healthcare accessibility and functional capacity rather than a direct determinant of maternal health outcomes. Preventive services were examined through immunization coverage. Health outcomes were assessed using the IMR and MMR, widely used indicators of health system performance. All indicators were selected for their sensitivity to changes in access, quality, and effectiveness of care and for their direct measurement or proxy representation of UHC coverage dimensions.

### 2.5 Statistical analysis

The analytical strategy was organized as a hierarchy of evidence, with segmented interrupted time series (ITS) regression as the primary method for evaluating whether the Aardram Mission’s implementation coincided with structural changes in mortality trajectories. Descriptive comparisons, correlation analysis, and multivariable regression served as supporting and exploratory approaches rather than independent tests of a policy effect, as all relied on the same underlying annual time series and were, therefore, not statistically independent of the ITS findings or of one another. Reporting followed established recommendations for interrupted time series studies (18), with complete specification of coefficients, standard errors, t-statistics, and *p*-values for all models. Data compilation, cleaning, descriptive statistics, and visualization were performed using Microsoft Excel. Inferential analyses were conducted using JASP (version 0.18.3; www.jasp-stats.org), an open-source statistical software platform.

#### 2.5.1 Stage 1—descriptive comparison

Pre- and post-policy period means were compared descriptively. Accordingly, Table 2 reports descriptive summary statistics only. Formal statistical inference regarding policy-associated changes is based exclusively on the segmented interrupted time series models described in Stage 2.

**Table 2:** Descriptive summary of UHC indicators before (2010–2016) and after (2017–2024) the implementation of the Aardram Mission.

Domain	Indicator	Pre-mean ± SD	Post mean ± SD	Mean diff
Financing	Health budget (₹ Cr)	4440.1 ± 1070.4	8849.8 ± 1233.4	4409.6
Financing	Insurance coverage (%)	20.4 ± 2.5	39.6 ± 4.6	19.2
Service delivery	OP visits (per 1,000)	48.7 ± 3.3	65.9 ± 6.1	17.2
Prevention	Immunization (%)	87.0 ± 2.2	91.8 ± 0.5	4.8
Outcomes	IMR (per 1,000)	12.1 ± 0.7	6.9 ± 1.8	−5.3
Outcomes	MMR (per 100 K)	70.1 ± 9.5	36.5 ± 7.4	−33.6

The descriptive summary indicates improvements across all six indicators during the post-policy period, including increased health expenditure, insurance coverage, and outpatient utilization; improved immunization coverage; and reductions in maternal and infant mortality. These observations provide descriptive context for the interrupted time series analysis presented below.

Source: Health budget data were obtained from Kerala State Budget documents, insurance coverage and OP visit data from Health at a Glance: Kerala reports, immunization coverage data from Health at a Glance: Kerala reports, and IMR and MMR data from Sample Registration System (SRS) reports. NFHS data were used for validation only. These findings are reported as descriptive comparisons and were not used for causal or primary inferential interpretation (see Section 2.5, Stage 1).

#### 2.5.2 Stage 2—interrupted time series regression (primary analysis)

Segmented regression within JASP’s general linear model framework was used to formally test whether the post-2017 trajectory departed from the pre-existing trend for each outcome (IMR, MMR, and immunization coverage), directly addressing the study’s central research question. The model incorporated (a) a continuous time variable, (b) a binary policy indicator, (c) a time × policy interaction term, and (d) an outcome variable, yielding simultaneous estimates of the pre-policy slope (*β*₁), the level change at implementation (*β*₂), and the post-policy slope change (*β*₃).

Before presenting the equation, it is useful to describe conceptually what segmented regression estimates. The model decomposes an observed annual outcome series into four components: The underlying pre-policy trend (how the outcome was already changing before 2017), an immediate level change at the point of implementation (a one-time shift coinciding with 2017), a change in slope thereafter (whether the rate of change itself accelerated or decelerated following implementation), and, by combining the first and third components, a counterfactual trajectory representing the outcome path that would plausibly have continued had the pre-2017 trend persisted unchanged. The gap between the observed post-2017 trajectory and this counterfactual is the quantity of primary analytical interest in this study.

The segmented regression equation took the following form:


$$\begin{array}{l} \text{Outcome}=\beta_{0}+\beta ₁(\text{Time})+\beta ₂(\text{Policy Indicator})\\ +\beta ₃(\text{Time}\times \text{Policy Indicator})+\varepsilon \end{array}$$

where *β*₁ represents the pre-policy trend, *β*₂ represents the level change, and *β*₃ represents the slope change coinciding with policy implementation (*n* = 15 annual observations, 2010–2024). The treatment-coinciding effect was decomposed as follows:


Effect(t)=Observed(t)−Counterfactual(t)=[β₂+β₃(t)]

representing, respectively, the immediate post-policy shift and the cumulative divergence in the outcome trajectory over time. This counterfactual framework is valid provided that (a) pre-policy trends are stable and approximately linear, (b) no other major confounding policy changes coincide precisely with 2017, and (c) unmeasured confounders are reasonably absorbed into the baseline trend. These are strong assumptions in ecological, single-group design, and their plausibility is discussed further in the Limitations section. Given the limited number of observations, autocorrelation diagnostics were essential, as prior short series ITS literature indicates that standard errors may be unreliable under temporal dependency (1, 20).

#### 2.5.3 Stage 3—sensitivity analysis

To assess the robustness of the segmented regression estimates, given the limited number of observations (*n* = 15) and the overlap of the post-policy period with the COVID-19 pandemic (2020–2022), two additional specifications were estimated. First, a COVID-adjusted model added a binary COVID indicator (coded 1 for 2020–2022 and 0 otherwise) as an additional covariate (*β*₄):


$$\begin{array}{l} \text{Outcome}=\beta_{0}+\beta ₁(\text{Time})+\beta ₂(\text{Policy})\\ +\beta ₃(\text{Time}\times \text{Policy})+\beta ₄(\text{COVID})+\varepsilon \end{array}$$

Second, a COVID-excluded model was estimated using a restricted sample omitting pandemic years (*N* = 12: 2010–2019, 2023–2024) to evaluate whether the core policy-coinciding effects (*β*₂, *β*₃) remained stable. Given the reduced sample size in all specifications, 95% confidence intervals are reported alongside point estimates and interpreted with appropriate caution, consistent with recommendations for short series ITS designs (21).

#### 2.5.4 Structural break test

To formally test whether 2017 coincided with a statistically detectable change in the underlying regression relationship—directly complementing the segmented regression described above—a Chow test (22) was performed at the predetermined 2017 break point for each outcome (IMR, MMR, and immunization coverage), comparing the residual sum of squares from a single pooled model against the sum of the residual sums of squares from separate pre- and post-policy sub-period models. The break date was treated as known *a priori*, corresponding to the documented policy implementation date, rather than statistically estimated, consistent with the study’s quasi-experimental design. The Chow test results are reported in Table 3 and discussed alongside the segmented regression findings in Section 3.

**Table 3:** Chow structural break test in 2017.

Outcome	*F*-statistic	*p*-value	Interpretation
IMR	*F* (2, 11) = 8.83	0.005	Statistically significant break; corroborates the segmented regression slope-change finding
MMR	*F* (2, 11) = 8.14	0.007	Statistically significant break; corroborates the segmented regression level−/slope-change finding
Immunization coverage	*F* (2,11) = 115.39	<0.001	Statistically significant break in the regression relationship, notwithstanding the non-significant individual *β*₂/*β*₃ terms in Table 7, reflecting the joint (rather than individual-coefficient) nature of the Chow test

The break date (2017) was treated as known a priori, corresponding to the documented policy implementation date, rather than statistically estimated. The Chow *F*-statistic tests the joint null hypothesis that the level-change and slope-change coefficients (*β*₂, *β*₃) are simultaneously zero, comparing a pooled single-trend model with the segmented model in Table 7/Section 2.5. Full computation details are provided in Supplementary Table S1.

#### 2.5.5 Stage 4—exploratory multivariable regression

Multivariable linear regression explored system-level associations between financing, service delivery, and mortality outcomes (Table 4). Given the anticipated high intercorrelation among health system indicators, reflecting their common origin in a single integrated reform package, these models are presented as hypothesis-generating rather than confirmatory, and their interpretation is addressed explicitly below.

**Table 4:** Regression model of health outcomes.

Model	Equation	*R* ^2^	Adj. *R*^2^	*F*-stat	*p*-value
IMR model	IMR = 17.301–0.00117 × Budget	0.959	0.955	303.48	<0.001
MMR model	MMR = 54.81–0.0143 × Budget + 0.174 × Insurance +1.538 × Visits	0.995	0.993	770	<0.001

*N* = 15 annual observations. Coefficients should be read as descriptive of a jointly collinear predictor set (see Table 9 and Section 2.5, Stage 4) rather than as independent effect estimates.

Multicollinearity among predictors was assessed using the variance inflation factor (VIF). Following O′Brien’s (20) caution against applying rigid VIF thresholds without context, values were interpreted relative to the conceptual structure of the model rather than as automatic disqualifying criteria. The four predictors examined (health budget, insurance coverage, outpatient visits, and FHC expansion) represent conceptually interrelated components of a single integrated reform package rather than independent policy levers; consequently, severe multicollinearity (VIF = 17.58–82.24) was anticipated rather than treated as a modeling failure. Removing conceptual central variables solely to minimize the VIF would compromise the substantive interpretability of the models relative to the reform’s actual content. As a robustness check, reduced-predictor specifications retaining only the least collinear variable in each model were estimated alongside the full models. Coefficient signs and general magnitudes were consistent between specifications (Supplementary Table S2), supporting the interpretation of the full-model associations as reflecting a coherent health system strengthening trend rather than an artifact of any single collinear predictor. Accordingly, all multivariable regression findings in this study are interpreted as exploratory descriptions of system-level associations rather than as evidence identifying independent, causally isolable determinants of mortality.

#### 2.5.6 Stage 5—correlation analysis

Pearson product–moment correlation coefficients were computed to assess linear associations between health system variables and health outcomes, with statistical significance assessed using two-tailed tests at *α* = 0.05 (Table 5). As all variables examined exhibited pronounced, largely monotonic trends over the 15-year study period, high correlation coefficients are an expected feature of any set of co-improving annual time series and are interpreted in this study as reflecting shared temporal trajectories rather than direct causal relationships between any single predictor and mortality reduction.

**Table 5:** Correlation between health system variables and outcomes.

Predictor	IMR (*r*)	MMR (*r*)	*p*-value
OP visits	−0.988	−0.972	<0.001
Health budget	−0.979	−0.992	<0.001
Insurance coverage	−0.978	−0.958	<0.001
FHCs	−0.983	−0.91	<0.001

Source: Health at a Glance: Kerala Health Report.

## 3 Results

The study presents empirical findings in the order established by the analytical hierarchy described in Section 2.5: Descriptive comparison, interrupted time series regression (primary analysis), sensitivity and structural break testing, and exploratory regression and correlation analyses.

### 3.1 Descriptive pre–post comparison

Table 2 presents descriptive pre- and post-policy period means for six UHC indicators. These comparisons are presented for descriptive context only, given that annual observations violate the independence assumption underlying standard inferential tests; formal inference regarding trend changes is provided by the segmented regression models described in Section 3.2.

Table 2 shows that all six indicators moved in the hypothesized direction between the pre- and post-policy periods. Health budget and insurance coverage roughly doubled, outpatient visits and immunization coverage increased, and both the IMR and MMR declined. These descriptive differences motivate, but do not by themselves establish, the formal trend analysis presented next.

### 3.2 Interrupted time series regression

Diagnostic testing (Table 6) confirmed the suitability of the annual time series data for ITS regression. Durbin–Watson, Breusch–Godfrey, and Ljung–Box tests indicated no statistically significant residual autocorrelation, and the augmented Dickey–Fuller (ADF) test indicated stationarity of all outcome variables (*p* < 0.05). Newey–West heteroscedasticity- and autocorrelation-consistent (HAC) standard errors were applied as an additional precaution. Residual-versus-fitted and residual-versus-time plots for all three ITS models are provided in Supplementary Figure S1 and show no evident patterns inconsistent with these diagnostic results. These tests and plots themselves have limited power in short series, a point revisited in the Limitations.

**Table 6:** Autocorrelation and stationarity diagnostic.

Test	IMR model	MMR model	Immunization model	Interpretation
Autocorrelation tests				
Durbin–Watson (DW)	1.89	1.92	1.87	Values near 2.0 indicate minimal autocorrelation
Breusch–Godfrey (*p*-value)	0.312	0.287	0.341	*p* > 0.05: No significant autocorrelation
Ljung–Box Q (*p*-value)	0.284	0.256	0.298	*p* > 0.05: No significant autocorrelation
Stationarity tests (ADF)				
ADF statistic	−3.84	−4.12	−2.91	
ADF *p*-value	0.018	0.009	0.048	*p* < 0.05: Stationarity (reject unit root)
Remedial measures				
Newey–West HAC SE applied	Yes	Yes	Yes	Precautionary correction for robust inference

DW values near 2.0 indicate minimal autocorrelation; Breusch–Godfrey and Ljung–Box tests with a *p*-value of >0.05 indicate no significant residual autocorrelation; the ADF test with a *p*-value of <0.05 indicates stationarity (rejection of a unit root). Residual diagnostic plots corresponding to these tests are provided in Supplementary Figure S1.

Table 7 presents the segmented regression coefficients for each outcome. Both the IMR and MMR exhibited statistically significant pre-policy downward trends (*β*₁), consistent with the pre-existing structural improvements described in Section 1, and the slope-change coefficients (*β*₃) were also statistically significant, indicating a change in the trajectory coinciding with the 2017 policy implementation. The high *R*^2^ values (0.912–0.979) across all models substantially reflect the strength of the underlying time trend common to these annual series and should not, on their own, be interpreted as evidence of causal validity; a model dominated by a strong deterministic trend will tend to produce high *R*^2^ values regardless of the specific mechanism driving that trend. This caveat applies throughout the interpretation of model fit in this study.

**Table 7:** Time trend and interrupted time series analysis.

Outcome	Parameter	Coefficient	SE	*t*-statistic	*p*-value
IMR (per 1,000 live births)					
	*β*₀ (intercept)	12.84	0.42	30.57	<0.001***
	*β*₁ (pre-policy slope)	−0.661	0.08	−8.26	<0.001***
	*β*₂ (level change at 2017)	−0.89	0.58	−1.53	0.142
	*β*₃ (post-2017 Slope change)	−0.97	0.11	−8.82	<0.001***
	Model fit	*R*^2^ = 0.979		*F* = 170.3	<0.001***
MMR (per 100,000 live births)					
	*β*₀ (intercept)	81.43	2.87	28.37	<0.001***
	*β*₁ (pre-policy slope)	−4.221	0.54	−7.82	<0.001***
	*β*₂ (level change at 2017)	−5.67	3.94	−1.44	0.168
	*β*₃ (post-2017 slope change)	−6.12	0.74	−8.27	<0.001***
	Model fit	*R*^2^ = 0.969		*F* = 459.0	<0.001***
Immunization coverage (%)					
	*β*₀ (intercept)	84.71	1.24	68.31	<0.001***
	*β*₁ (pre-policy slope)	0.38	0.29	1.31	0.216
	*β*₂ (level change at 2017)	1.82	1.71	1.06	0.311
	*β*₃ (post-2017 slope change)	0.21	0.39	0.54	0.601
	Model fit	*R*^2^ = 0.912		*F* = 38.21	<0.001***

*N* = 15 annual observations (2010–2024); pre-policy *n* = 7 years (2010–2016); post-policy *n* = 8 years (2017–2024).

Interpreting these coefficients descriptively, the IMR exhibited a baseline declining trend of 0.661 per 1,000 live births annually before 2017, while the MMR declined at a rate of 4.221 per 100,000 live births annually, consistent with pre-existing structural improvements in the health system. The slope-change coefficients indicate that the rate of the IMR decline coinciding with policy implementation was approximately 2.46 times the pre-policy rate. Immunization coverage, which was already close to ceiling levels before policy implementation, showed no statistically significant level or slope change, consistent with a prevention program that was already well established before 2017 (see Section 4.4).

### 3.3 Sensitivity analysis and structural break test

Sensitivity analyses (Table 8) assessed the robustness of the segmented regression estimates to COVID-19 confounding. The slope-change coefficient (*β*₃) for the IMR remained statistically significant across all three specifications (full model: −0.417, *p* < 0.05; COVID-adjusted model: −0.405, *p* < 0.05; COVID-excluded model: −1.114, *p* < 0.05), and the COVID indicator itself was non-significant (*β*₄ = −0.314, *p* > 0.05), suggesting the pandemic did not independently disrupt the IMR trajectory beyond the ongoing policy-coinciding trend. The immunization coverage results were similarly stable across specifications.

**Table 8:** COVID-19 sensitivity analysis.

Outcome	Parameter	Full model (*N* = 15)	COVID-adjusted (*N* = 15)	COVID-excluded (*N* = 12)
IMR (per 1,000 live births)				
	*β*₀ (intercept)	13.00 (0.34)	13.00 (0.34)	13.00 (0.32)
	*β*₁ (pre-policy slope)	−0.286 (0.094)	−0.286 (0.095)	−0.286 (0.089)
	*β₂* (level change 2017)	1.25 (0.89)	1.25 (0.90)	6.80 (1.39)**
	*β*₃ (post-2017 slope change)	−0.417 (0.121)*	−0.405 (0.124)*	−1.114 (0.173)*
	*β*₄ (COVID effect)	—	−0.314 (0.374)	—
	*R* ^2^	0.979	0.98	0.981
MMR (per 100,000 live births)				
	*β*₀ (intercept)	83.11 (1.65)	83.11 (1.52)	83.11 (1.82)
	*β*₁ (pre-policy slope)	−4.321 (0.459)***	−4.321 (0.421)***	−4.321 (0.503)***
	*β*₂ (level change 2017)	−16.86 (4.35)*	−16.86 (4.00)**	−1.51 (7.89)
	*β*₃ (post-2017 slope change)	1.488 (0.592)*	1.591 (0.547)	−0.479 (0.981)
	*β*₄ (COVID effect)	—	−2.882 (1.651)	—
	*R* ^2^	0.987	0.99	0.985
Immunization coverage (%)				
	*β*₀ (intercept)	84.00 (0.16)	84.00 (0.15)	84.00 (0.13)
	*β*₁ (pre-policy slope)	1.000 (0.046)***	1.000 (0.042)***	1.000 (0.037)***
	*β*₂ (level change 2017)	6.25 (0.43)***	6.25 (0.39)***	4.90 (0.57)***
	*β*₃ (Post-2017 slope change)	−0.857 (0.059)*	−0.868 (0.054)*	−0.700 (0.071)*
	*β*₄ (COVID effect)	—	0.294 (0.163)	—
	*R* ^2^	0.994	0.996	0.997

Standard errors are reported in parentheses; *p < 0.05, **p < 0.01. Full coefficient tables, including intercepts and pre-policy slopes, are provided in the original model output.

The MMR results were less robust across model specifications. While the level-change coefficient (*β*₂) was significant in the full and COVID-adjusted models (−16.86, *p* < 0.05, and *p* < 0.01, respectively), it lost statistical significance when COVID-period years were excluded (*β*₂ = −1.51, *p* > 0.05, *N* = 12), and the slope-change coefficient (*β*₃) was not significant in any specification. This indicates that part of the estimated immediate post-policy shift in the MMR may be sensitive to the inclusion of COVID-period observations and to the reduced statistical power associated with the smaller restricted sample. This finding is treated as a limitation of the MMR-specific ITS estimates rather than as a robust, independent policy-coinciding effect (see Section 4.7 and Limitations).

### 3.4 Exploratory multivariable regression

Table 4 presents exploratory multivariable regression models describing associations between health budget, insurance coverage, outpatient visits, and mortality outcomes. These models were undertaken to describe exploratory system-level associations and were not intended to identify independent, causally isolable determinants of mortality. Both the IMR model (budget alone) and the MMR model (budget, insurance coverage, and outpatient visits) achieved high explanatory power (*R*^2^ > 0.95); however, as the corresponding VIF diagnostics in Table 9 confirm, these predictors are highly collinear, and the results should be interpreted as reflecting the overlapping trajectory of an integrated set of health system reforms rather than the independent, separable contribution of any single variable.

**Table 9:** Variance inflation factor (VIF) diagnostics for multiple regression models.

Outcome model	Predictor	Coefficient	SE	*t*-statistic	*p*-value	VIF
IMR model	Health budget	−0.00117	6.73 × 10^−5^	−17.42	<0.001	N/A
MMR model	Health budget	−0.01428	0.00142	−10.03	<0.001	82.24
MMR model	Insurance coverage	0.17384	0.15809	1.1	0.295	17.58
MMR model	Outpatient visits	1.53836	0.31814	4.84	<0.001	65.03

Interpreted according to O’Brien (20) within the context of a conceptually integrated predictor set rather than as an automatic disqualifying criterion (see Section 2.5, Stage 4). A reduced-predictor robustness check is reported in Supplementary Table S2.

### 3.5 Correlation analysis

Table 5 presents Pearson correlation coefficients between four health system variables and the two mortality outcomes.

As all four predictor variables and both outcome variables exhibited pronounced, largely monotonic trends over the 15-year study period, these near-unity correlation coefficients are an expected feature of any set of co-improving time series and should be interpreted as reflecting shared temporal trajectories rather than direct causal relationships between any single predictor and mortality reduction. Outpatient visits and health budget showed the strongest correlation overall, while FHC expansion correlated more strongly with the IMR than with the MMR; these differences are noted descriptively and are not evidence of differential causal mechanisms.

### 3.6 UHC progress assessment against reference benchmarks

Table 10 presents an indicative, author-constructed comparison of Kerala’s UHC progress with illustrative reference targets informed by WHO UHC monitoring literature and contextualized within Kerala’s public-provision-oriented model. As Kerala’s system differs structurally from the insurance-financed systems that motivate many international UHC benchmarks, this table should be read as a locally contextualized gap analysis rather than a formal WHO-certified scorecard.

**Table 10:** UHC progress assessment against illustrative reference targets, contextualized within Kerala’s public-provision-oriented model.

Dimension	Current	Target	Achievement (%)	Gap (%)	Severity
Financial protection	42%	90%	46.7	53.3	Critical
Service coverage	74%	85+	87.1	12.9	Moderate
Infrastructure	80%	100%	80	20	Moderate
Prevention	92%	95%	96.8	3.2	Low

Achievement (%) calculated as (Current/Target) × 100; Gap (%) = 100 − Achievement.

Kerala’s progress across four illustrative Universal Health Coverage dimensions is presented, including current values, reference targets, achievement, remaining gaps, and severity. Financial protection showed the largest gap, while prevention was closest to the target.

Using insurance coverage as a proxy for financial protection, this dimension showed the largest gap relative to the illustrative reference target. However, insurance enrollment does not directly measure catastrophic health expenditure or impoverishment. Kerala’s public health system may, therefore, provide greater financial protection than enrollment alone indicates, reflecting its longstanding reliance on publicly financed primary care rather than insurance-based financing. After correction of the service coverage calculation, service coverage and infrastructure showed smaller gaps, whereas prevention indicators performed best because of sustained immunization and disease prevention programs. Quality of care was not assessed against standardized WHO benchmarks and remains an important area for future research.

## 4 Discussion

The Aardram Mission’s implementation coincided with a shift toward health system strengthening through the transformation of PHCs into FHCs (23). This aligns with the global view of primary health care as central to advancing UHC. The policy’s architecture encompasses financial protection, service coverage, and prevention integration—dimensions widely recognized as determinants of maternal and neonatal health outcomes (24, 25). The findings reported here, showing declines in the IMR and MMR coinciding with policy implementation, are consistent with the broader literature on structured health system reform as a plausible contributor to accelerated progress beyond secular trends (26, 27), while the study’s design limits how strongly this contribution can be asserted.

### 4.1 Health system reform as a contributing factor in UHC Progress

The Aardram Mission’s transformation of PHCs into FHCs parallels similar transformations noted globally. In China, the primary health care reform initiated in 2009 was associated with increased primary care utilization (28). In Bangladesh, a multi-sectoral approach demonstrated that structured programming combined with community engagement was associated with maternal mortality reductions (29). The Aardram Mission shows parallel improvements in financial protection, service delivery, and health outcomes, a pattern consistent with the broader literature suggesting that integrated health system strengthening—rather than vertical, single-issue interventions—may produce synergistic associations beyond those expected from individual program components in isolation (15). This remains an association observed across countries rather than a demonstrated causal mechanism specific to Kerala.

### 4.2 Financial protection and health financing mechanisms

The expansion of insurance coverage from 20.4 to 39.6% coincides with a period of reduced barriers to service utilization, yet insurance enrollment is only one component of financial protection. Kerala-specific household survey evidence indicates that out-of-pocket expenditure and hospitalization-related financial burden remain substantial even among insured populations (30), reinforcing the need to interpret the insurance-based proxy cautiously. Direct measures of catastrophic expenditure and impoverishment were unavailable as continuous annual administrative data (Section 2.3) and represent an important direction for future research using data from the NSS Health Consumption Expenditure rounds. Nevertheless, the expansion of health insurance coverage has been recognized as a contributing factor to improved maternal and reproductive health service utilization across LMICs (4). The exploratory regression model (Table 4) indicated that insurance coverage was not independently associated with maternal mortality after adjustment for health budget and outpatient visits (*β* = 0.174, SE = 0.158, *t* = 1.10, *p* = 0.295). The severe multicollinearity among these conceptually related predictors (VIF = 17.58; Table 9) suggests that the observed associations should be interpreted as reflecting independent components of an integrated health system reform rather than independent effects of individual variables (27).

The observed insurance coverage expansion reflects the combined effect of multiple concurrent initiatives rather than the Aardram Mission alone. While the Aardram Mission (2017) integrated financial protection mechanisms into primary care strengthening, the nationally launched Ayushman Bharat PM-JAY scheme (September 2018), the pre-existing Rashtriya Swasthya Bima Yojana (RSBY), and Kerala’s state-specific Karunya Arogya Suraksha Padhathi (KASP) also contributed to insurance coverage expansion during this period. The ITS estimates for insurance coverage should, therefore, be interpreted as reflecting the cumulative effect of these overlapping interventions rather than an isolated Aardram Mission association. Insurance coverage alone does not resolve financial burden: Implementation research from Indonesia shows that national insurance affects primary care performance unevenly without complementary payment reforms (31), and evidence from the Middle East and North Africa indicates that health expenditure per capita is associated with MMR and IMR reductions more broadly (32). Governance factors, such as strategic vision, community engagement, and transparency, remain important influences on whether policy strategies translate into intended UHC outcomes (33); even insured populations may continue to incur out-of-pocket expenditures depending on frontline facility practice (6).

Benchmarked against the illustrative reference target presented in Table 10, Kerala’s insurance coverage gap should not be read as UHC failure. Kerala’s healthcare model differs fundamentally from insurance-driven systems, emphasizing publicly funded service provision over insurance-based expansion (34). The multidimensional nature of UHC means that lower insurance coverage does not necessarily equate to inadequate financial protection (30). Further insurance expansion would represent a significant fiscal commitment that must be weighed against Kerala’s available budgetary resources and competing health system priorities (35, 36); the identified gap is, therefore, better framed as a policy priority area warranting strategic evaluation of cost-effectiveness relative to the state’s existing public-provision model, rather than as evidence of policy failure (34). Future policy refinements should focus on strengthening the public-provision model by addressing residual out-of-pocket expenditure barriers through targeted interventions (30).

### 4.3 Service delivery expansion and primary health care utilization

The transformation of PHCs into FHCs and the expansion of outpatient services indicate substantial strengthening of the primary health care infrastructure. This is consistent with evidence that improvements in service delivery contribute to better population health outcomes, including lower mortality (27). Similar reforms in Ethiopia improved infrastructure and service delivery, although inequities remained in underserved areas (19). In Brazil, the Family Health Strategy was associated with reductions in neonatal and post-neonatal mortality independent of socioeconomic conditions (37, 38). Community health worker programs in South Asia have also been linked to improved healthcare access and health outcomes (39, 40).

### 4.4 Immunization and prevention integration: reducing preventable disease burden

Immunization coverage (Table 2) was close to the illustrative reference target, indicating strong performance of preventive services. This may reflect the integration of preventive care into the FHC model, as integrated PHC is generally more effective and sustained than vertical programs (41). Similar evidence from China suggests that improvements in education, income, and living conditions strengthen the association of preventive interventions for mortality (42). In Pakistan, family planning programs have also contributed to reducing high-risk pregnancies. However, lower access among rural and indigenous populations indicates that improvements in service coverage have not been equally distributed (43).

### 4.5 Maternal and infant mortality reduction

The 47.9% decline in the MMR and 43.8% decline in the IMR observed over the post-policy period are broadly consistent with international evidence on health system-associated mortality reductions. India’s national experience with maternal and neonatal mortality reduction shows that gains in the coverage of antenatal care, facility delivery, and emergency obstetric care were associated with the NRHM (2005–2012), providing a precedent that Kerala’s Aardram Mission framework broadly parallels. The time series in this study showed declines in the IMR and MMR coinciding with policy implementation; however, this association appears to be part of a multiplicative combination of factors rather than a single additive policy effect (27). Indonesia’s Saber program achieved reductions in the MMR (30%) and IMR (25%) through the integration of early detection systems, rapid reporting, and community capacity building, illustrating that multi-component approaches can produce synergistic associations (44). Malawi’s Mai Mwana Project similarly combined women’s groups with infant care support to improve care-seeking practices and reduce mortality (45). By contrast, Nepal’s progress in reducing maternal mortality stalled despite policy presence, with weak implementation, poor quality of care, and barriers faced by marginalized groups illustrating that policy presence alone does not guarantee policy success (46); evidence from Angola similarly shows that expanding access without corresponding attention to quality yields limited benefits (47). These contrasting cases underscore that the Kerala findings should be interpreted as being consistent with, rather than proof of, an effective implementation process.

Beyond primary health care strengthening, Kerala’s historical health achievements reflect many factors that predate the Aardram Mission by decades, including sustained investment in women’s education—one of the most consistent determinants of maternal and infant health across epidemiological studies. This is consistent with the broader “Kerala model” literature, which attributes the state’s early demographic transition to land reform, redistributive social policy, political mobilization, and sustained public investment in education and health since the mid-20th century (16). Comparable evidence from China shows that social factors including income, education, and living conditions fundamentally influence mortality reduction (34). Improvements in water and sanitation infrastructure implemented across Kerala over several decades have likewise directly contributed to reductions in infectious disease burden and improvements in child survival. Women’s empowerment initiatives, labor participation, social security programs, and political representation were established well before the Aardram Mission and plausibly created enabling environments for health-seeking behavior and family planning adoption (34). Consequently, the accelerated decline in mortality identified in the ITS analysis is best interpreted as the Aardram Mission’s marginal contribution within an already favorable structural context, rather than as evidence that primary care reform alone explains Kerala’s health outcomes.

#### 4.5.1 Equity disparities in health outcomes and service access

Despite the aggregate improvements documented, substantial equity gaps persist across geographic, socioeconomic, and gender dimensions (34). Rural and indigenous populations continue to experience lower access to immunization and prevention care despite policy expansion (17). Socioeconomic inequalities constrain facility utilization, as out-of-pocket health expenditure burdens remain concentrated among low-income households. Financial protection remains critically limited relative to the illustrative reference target, disproportionately affecting lower-income populations who face the greatest out-of-pocket barriers (30). The intersection of rural residence, low household income, and female sex creates compounding disadvantages in healthcare access, representing a critical equity frontier for Kerala’s health system (34).

### 4.6 Health system determinants and sustainability

The very high correlations between health system variables and mortality (r = −0.910 to −0.992; Table 5), together with VIF diagnostics confirming severe multicollinearity (VIF = 17.58–82.24; Table 9), indicate that these variables function as interdependent components of an integrated reform package rather than as independent, separable determinants. Consequently, the multivariable regression findings of this study are interpreted as exploratory evidence of co-occurring system-level trends, underscoring the value of an integrated policy approach (27) rather than the isolated contribution of any single level. Governance is a recognized enabler of reform effectiveness, supported by evidence from Tanzania, while UHC progress more broadly depends on simultaneous improvements in access, quality, and financial protection, as observed in comparative evidence from South Asia (2). Although financing for comprehensive primary care appears feasible within existing commitments in India (48), systemic barriers such as fragmentation and weak coordination persist (49), with digital systems offering supportive but conditional gains (50). Persistent inequities highlight that UHC remains incomplete without targeted interventions (14), and global evidence links underfunding and poor quality of care to broader system inefficiencies (51, 52). In this context, the Aardram Mission demonstrates a comprehensive reform approach whose implementation coincided with an accelerated decline in mortality, although remaining gaps—particularly in financial protection—warrant continued attention to equity and quality to ensure sustainability.

### 4.7 COVID-19 pandemic as a concurrent health system shock

Interpretation of post-policy trends must be contextualized within the COVID-19 pandemic, which coincided with the consolidated phase of Aardram Mission reforms (2020–2022). Global evidence documents unprecedented pressure on health systems during this period, with consequences for healthcare-seeking behavior, service capacity, and routine health program functioning (53, 54). Kerala’s health system faced staff deployment toward COVID-19 response activities and potential disruption to routine outpatient services during this period (55); consequently, some observed changes in utilization patterns and health outcomes during this window may reflect pandemic-related dynamics as much as the effects of policy implementation. Kerala’s relatively strong institutional capacity and coordination during the pandemic may have partially offset service disruptions relative to other Indian states (56), and sustained government investment in maternal health programs during this period appears to have provided support to pregnant women and newborns (57), although this remains a plausible contextual explanation rather than a formally tested mechanism.

The formal sensitivity analyses reported in Section 3.3 (Table 8) indicate that the IMR and immunization coverage findings were robust to COVID-19 adjustment, with the pandemic indicator itself non-significant in both models. However, the MMR level-change estimate was sensitive to the exclusion of pandemic years, losing statistical significance in the COVID-excluded specification. This suggests that part of the estimated immediate post-2017 shift in maternal mortality may reflect pandemic-related dynamics or reporting changes rather than the effects of the Aardram Mission alone, and this estimate should be interpreted with greater caution than the corresponding IMR and immunization results.

## 5 Policy priorities and implementation framework

The Aardram Mission’s implementation coincided with meaningful mortality trend changes, but persistent gaps in financial protection and service quality point toward several priority areas for policy attention, informed by the study’s findings: (1) exploring integrated financing mechanisms—such as capitation-based purchasing models linking insurance more directly to comprehensive primary care—with a focus on reducing out-of-pocket expenditure and strengthening protection against catastrophic health costs through regular monitoring (9, 14); (2) strengthening governance through district-level health accountability structures with regular performance review and transparent health management information systems (58–60); (3) addressing workforce gaps through staffing norms, career progression pathways, and comprehensive training with supportive supervision (58, 61, 62); (4) prioritizing equity through district-level equity audits examining rural–urban gaps, socioeconomic gradients in service access and expenditure burden, and gender-disaggregated outcomes, along with community health worker integration targeting marginalized populations (4, 63, 64); (5) supporting immunization performance beyond current plateaus through integrated service delivery and equity-disaggregated monitoring (14); (6) considering institutional strengthening through a dedicated state health resource center for technical capacity and adaptive management (33, 59, 65); and (7) establishing structured policy learning mechanisms through periodic evaluation and policy dialogue to inform adaptation and potential scaling to other states (14).

Implementation of these priorities would benefit from a phased approach: An initial foundation-setting phase establishing financing mechanisms, governance structures, and institutional capacity (59); a subsequent systems-operationalization phase focused on completing staff training, equity assessments, and health information system deployment (61, 62); and a longer-term phase focused on full operationalization, rigorous policy change evaluation, and evidence generation for potential scaling (10, 14). Cross-cutting enablers for these recommendations include sustained political commitment, diversified financing mechanisms, and institutional capacity developed through academic and technical partnerships (58, 61). An equity-focused approach—incorporating disaggregated targets for vulnerable groups, evidence-based decision-making, and the involvement of communities and frontline workers in planning—together with transparent reporting and flexible management for course correction, is likely to be more sustainable than short-term, narrowly targeted interventions (1, 4). These recommendations are offered as an evidence-informed starting point for discussion among Kerala’s health policy stakeholders, rather than as a definitive or exhaustive roadmap; further research—including comparator analysis and refined financial protection measures identified in the Limitations below—would strengthen the evidence base underpinning these recommendations (66).

## 6 Conclusion

The Aardram Mission’s implementation coincided with meaningful improvements in mortality trends in Kerala, consistent with the broader literature suggesting that targeted primary care strengthening can be associated with accelerated declines in maternal and infant mortality (10, 14, 68). This coincided with expanded health insurance enrollment, representing progress consistent with movement toward UHC within India’s broader health system transformation (2). However, this study’s findings indicate that improvements in mortality trends alone do not constitute comprehensive UHC implementation: Persistent challenges and gaps remain in financial protection, alongside uneven service delivery (7, 9). These findings underscore an important distinction between policy-coinciding health improvements and system-wide UHC progress. While the Aardram Mission’s implementation coincided with positive health trends, implementation barriers and financing constraints appear to limit the extent to which the full spectrum of UHC has been achieved (6).

This study’s 15 annual observations (seven pre-policy and eight post-policy) were at or below the thresholds identified in the methodological literature as necessary for adequate statistical power in segmented regression (see Limitations); non-significant coefficients may, therefore, reflect limited statistical power rather than a true absence of effect. The evidence presented here suggests that primary care strengthening is likely to require complementary investment in health financing reform, governance strengthening, and equity-focused implementation strategies to close remaining UHC gaps (19, 33). Kerala’s experience illustrates that even health systems with favorable mortality indicators may require substantial structural reform to achieve financial protection and equitable access. Future policy attention could usefully focus on out-of-pocket payment barriers and implementation capacity at the district and facility level, with the aim of ensuring that UHC-related benefits reach disadvantaged populations more equitably. As the study period (2017–2024) overlaps with multiple concurrent health financing policy initiatives, including Ayushman Bharat PM-JAY, the observed insurance coverage expansion should be understood as reflecting the cumulative effect of overlapping interventions rather than an isolated Aardram Mission association.

## 7 Limitations

This study has several limitations. First, no contemporaneous comparator series was available; therefore, the single-group ITS design cannot exclude alternative explanations for the observed mortality decline, including concurrent state or national reforms and secular trends unrelated to the Aardram Mission. Comparing Kerala’s trajectory against other states or the national SRS trend would strengthen future causal inference. Second, the 15 annual observations (seven pre-policy and eight post-policy) were at or below the thresholds identified in the methodological literature as necessary for adequate power in segmented regression, particularly for level-change effects (*β*₂) (67); non-significant coefficients (e.g., the immunization ITS terms and the MMR slope-change term) may reflect low power rather than a true absence of effect, and wide confidence intervals warrant caution against strong interpretation. Third, the study period overlaps with several concurrent financing interventions—Ayushman Bharat PM-JAY (2018), the pre-existing RSBY, and Kerala’s own KASP—so observed insurance and mortality trends reflect their cumulative effect and cannot be attributed to the Aardram Mission alone. Fourth, the post-policy period overlaps COVID-19 (2020–2022); sensitivity analyses (Table 8) showed the IMR and immunization findings were robust to COVID-19 adjustment, but the MMR level-change estimate lost significance when pandemic years were excluded and should therefore be interpreted with corresponding caution. Fifth, insurance coverage was used as a proxy for financial protection; however, it does not measure catastrophic health expenditure or impoverishment, which are core WHO financial protection indicators because continuous annual data for these indicators are unavailable. Addressing this evidence gap should be a priority for future research using National Sample Survey (NSS) data. Sixth, all data were state-aggregated, precluding household-level inference regarding which populations experienced genuine financial protection. Seventh, the high *R*^2^ values and near-unity correlations reported throughout (Tables 4, 5, 7, 9) largely reflect shared secular trends across indicators and should not be interpreted as evidence of independent causal contributions from any single predictor. Finally, while Durbin–Watson, Breusch–Godfrey, Ljung–Box, and ADF tests (Table 6) showed no evidence of significant autocorrelation and confirmed stationarity, with Newey–West HAC errors applied as a precaution, these tests have limited power in short series and should be interpreted as supportive rather than conclusive.
