# Supplementary material for: Kerala’s Aardram Mission and universal health coverage: integrated time series evidence on mortality reduction and assessment of financial protection and equity gaps
Source: Front Public Health. 2026 Aug 5;14:1882081. doi: 10.3389/fpubh.2026.1882081 (PMC13485955; doi:10.3389/fpubh.2026.1882081)
Supplement: Supplementary file 1 [file Data_Sheet_1.PDF]

SUPPLEMENTARY MATERIAL

Kerala's Aardram Mission and Universal Health Coverage: Interrupted Time Series Evidence on Mortality Trends and Assessment of Financial Protection and Equity Gaps

Shahana K P [1], Balamurugan J [2*]

[1] Vellore Institute of Technology, Research Scholar, School of Social Science & Languages, Vellore, India.

[2] Vellore Institute of Technology, Research Scholar, School of Social Science & Languages, Vellore, India.

** Correspondence: Balamurugan J — balamurugan.j@vit.ac.in*

Supplementary Details

This supplementary material documents (a) all variables used in the interrupted time series analysis, including their operational definitions, measurement units, data sources, available years, and analytical role; (b) full computational detail for the Chow structural break test referenced in the main text (Table 3c); (c) a multicollinearity robustness check for the exploratory multivariable regression models; and (d) residual diagnostic plots for the three segmented regression models. It is provided to enhance transparency and reproducibility. All data represent annual state-level observations for Kerala (2010–2024; N = 15).

Supplementary Table S1: Variable Definitions, Sources, Measurement Units, and Data Availability (2010–2024)

This table provides comprehensive documentation of all variables used in the interrupted time series analysis of the Aardram Mission (2017) in Kerala. It specifies variable definitions, data sources, measurement units, temporal availability, and data harmonization procedures. All data are aggregated at the state (Kerala) level across the study period 2010–2024 (N=15 annual observations).

Variable	Operational Definition	Data Source	Unit	Years	Data Type & Frequency	Validation / Harmonization
Infant Mortality Rate (IMR)	Deaths of children under age 1 per 1,000 live births; primary outcome for child survival.	Sample Registration System (SRS) Statistical Reports; Office of the Registrar General and Census Commissioner, India	Per 1,000 live births	2010–2024	Secondary administrative; annual state-level aggregates	SRS primary source; cross-validated against DHS estimates for 2015–16 and 2019–21; no interpolation applied.
Maternal Mortality Ratio (MMR)	Maternal deaths per 100,000 live births; primary outcome for maternal health and policy effectiveness.	SRS Statistical Reports; Office of the Registrar General and Census Commissioner, India	Per 100,000 live births	2010–2024	Secondary administrative; annual state-level aggregates	SRS primary source; cross-validated against NFHS rounds 4/5 (2015–16, 2019–21); no imputation or extrapolation.
Immunization Coverage (Full Immunization)	% of children aged 12–23 months receiving all recommended vaccines; secondary outcome reflecting preventive service quality and reach.	Health at a Glance: Kerala annual reports (validated against NFHS rounds 4/5)	%	2010–2024	Secondary administrative and survey data; annual coverage rates	Cross-validated against NFHS survey data for 2015–16 and 2019–21 to confirm plausibility and reduce reporting bias; no interpolation.

Health Budget Allocation (State)	Total annual government expenditure on healthcare in Kerala (₹ crores), including PHCs, FHCs, taluk/district hospitals, and state-level programs; reflects system-level investment capacity.	Kerala State Budget Documents; Ministry of Health, Government of Kerala	₹ crores	2010–2024	Primary administrative fiscal data; annual budget records	Checked against government health department annual reports for consistency; nominal values retained (no inflation adjustment).
Insurance Coverage (Publicly Funded)	% of population enrolled in publicly funded health insurance schemes (incl. Ayushman Bharat PM-JAY and state schemes); enrolment proxy, not realized financial protection.	Health at a Glance: Kerala annual reports (validated against NFHS rounds 4/5)	% of population	2010–2024	Secondary administrative and survey data; annual coverage rates	Cross-referenced against NFHS rounds 4/5; does not measure catastrophic expenditure, OOP burden reduction, or realized financial protection (continuous annual data unavailable at required frequency).
Family Health Centres (FHCs) – Expansion Count	Cumulative number of PHCs/Sub-Health Centres transformed into FHCs under the Aardram Mission; reflects coverage of primary care restructuring.	Government Administrative Records (Dept. of Health Services, Govt. of Kerala); Aardram Mission implementation progress reports	Count (facilities)	2010–2024	Primary administrative data; annual facility count aggregates	Cross-checked against published Aardram Mission progress reports and state health department bulletins; count data, no imputation.
Outpatient Visits (State-level)	Total annual outpatient consultations across public sector facilities (PHCs, FHCs, taluk and district hospitals); measures service utilization and access.	Health at a Glance: Kerala annual reports (administrative compilation)	Count and per 1,000 population	2010–2024	Secondary administrative; annual facility utilization aggregates	Compiled from facility administrative reporting; no imputation/interpolation; rates derived using Census/SRS population estimates.
Policy Implementation Indicator (Binary)	Coded 0 for pre-policy (2010–2016) and 1 for post-Aardram Mission period (2017–2024); operationalizes intervention timing in segmented regression.	Study design; Government of Kerala notification of Aardram Mission launch (2017)	0/1	N/A (temporal index)	Derived analytical variable; created for time series partitioning	Policy launch date verified from official Government of Kerala documentation and gazette notifications; n=7 pre-policy, n=8 post-policy years.
COVID-19 Pandemic Indicator (Sensitivity Analysis)	Coded 1 for pandemic years (2020–2022), 0 otherwise; used in sensitivity analysis for potential pandemic confounding.	Study design; WHO and Government of India COVID-19 timeline documentation	0/1	N/A (temporal index)	Derived analytical variable; created for sensitivity specification	Pandemic period identified from official Government of India declarations and case-data timelines; used as covariate in COVID-adjusted ITS model (Section 2.5, Stage 3).
Time (Linear Trend)	Continuous annual time variable (coded 1–15 for years 2010–2024); represents secular trend and enables detection of policy-coinciding acceleration via segmented regression.	Study design (mathematical construct)	Years (ordinal, 1–15)	N/A (temporal index)	Derived analytical variable; continuous time encoding for regression	Constructed deterministically from calendar years 2010–2024; verified against observation sequence for consistency.

Notes on Data Quality and Harmonization

1. Study Period: All analyses use continuous annual time series data spanning 2010–2024 (N=15 observations), partitioned into pre-policy (2010–2016, n=7 years) and post-policy (2017–2024, n=8 years) phases for interrupted time series regression.

2. Primary Data Sources: Longitudinal dataset constructed from three core secondary administrative sources: (a) Health at a Glance: Kerala annual reports (service coverage, immunization, insurance, outpatient visits); (b) Kerala State Budget Documents (health budget allocations); (c) Sample Registration System (SRS) Statistical Reports (IMR and MMR). All sources provide continuous annual observations suitable for segmented regression without interpolation.

3. Supplementary Validation Data: NFHS (rounds 4 and 5) and National Sample Survey (NSS) datasets, collected at irregular intervals, were used exclusively for validation. Administrative estimates for IMR, MMR, immunization coverage, and insurance enrolment were cross-checked against survey-based estimates for corresponding years (2015–16 NFHS Round 4; 2019–21 NFHS Round 5) to confirm plausibility. No interpolation, extrapolation, or imputation was applied to the series used in statistical modelling.

4. Data Aggregation: All variables aggregated at Kerala state level into a single annual time series. No district-level or sub-regional stratification applied; analyses represent state-wide policy association.

5. Proxy Indicator Note (Insurance Coverage): Insurance coverage is used as a proxy for financial protection enrolment rather than as a direct measure of realized financial protection. The WHO UHC financial protection framework encompasses catastrophic health expenditure, impoverishment due to health spending, and out-of-pocket expenditure burden reduction. Continuous annual data for these measures at the required frequency were not available; insurance enrolment should be interpreted as indicating expansion of risk-pooling and population coverage rather than confirmation of achieved financial protection.

6. Missing Data: No data imputation or missing-value procedures were necessary. All selected indicators have continuous annual observations throughout 2010–2024 from their respective primary sources.

7. Temporal Validity: Pre-2017 secular trends (β_1) derived from 7 pre-policy years; post-policy slopes (β_3) estimated from 8 post-policy years. This partitioning ensures sufficient observations for trend estimation while maintaining analytical precision appropriate to short, interrupted time series designs (N=15; see main text Limitations).

8. Statistical Diagnostics: Autocorrelation assessed via Durbin-Watson (range: 1.87–1.92), Breusch-Godfrey, and Ljung-Box tests (all $p>0.05$). Stationarity confirmed via Augmented Dickey-Fuller test ($p<0.05$); no unit roots detected. Newey-West HAC standard errors applied for robustness. See main text Table 2 for full diagnostic results, and Supplementary Figure S1 (below) for corresponding residual plots.

9. Reproducibility: All analyses conducted in JASP statistical software (version 0.18.3). Raw data compilation and descriptive statistics performed in Microsoft Excel. Complete model specifications including coefficients, standard errors, t-statistics, and p-values are reported in the Results section tables. Reporting follows recommendations for interrupted time series studies (Jandoc et al., 2015; Bernal et al., 2017).

Supplementary Table S2: Chow Structural Break Test — Full Computation Detail

The Chow test (Chow, 1960) formally evaluates whether the 2017 policy implementation date coincides with a statistically detectable change in the regression relationship, by comparing a restricted (pooled, no-break) model against the unrestricted segmented regression model reported in the main text (Table 3a). The restricted model regresses each outcome on a single continuous time trend only (Outcome = $\beta_0 + \beta_1 \cdot$Time); the unrestricted model adds the policy-level and policy-interaction terms (Outcome = $\beta_0 + \beta_1 \cdot$Time + $\beta_2 \cdot$Policy + $\beta_3 \cdot$Time-since-Policy), matching the specification used throughout Section 2.5 and Table 3(a)/3(b) of the main text. The F-statistic is computed as:

$$F = [(RSS_r - RSS_u) / (k_u - k_r)] / [RSS_u / (n - k_u)]$$

where RSS_r and RSS_u are the residual sums of squares from the restricted and unrestricted models respectively, $k_r = 2$ and $k_u = 4$ are the number of estimated parameters in each model, and $n = 15$.

Table S2. Chow Test — Residual Sums of Squares and F-Statistics

Outcome	RSS (restricted)	RSS (unrestricted)	df1	df2	F-statistic (p-value)
IMR	7.101	2.726	2	11	8.826 (p = 0.0052)
MMR	160.671	64.798	2	11	8.138 (p = 0.0068)
Immunization Coverage	14.130	0.643	2	11	115.388 (p < 0.0001)

All three outcomes show a statistically significant structural break at 2017 ($p < 0.01$ in all cases), corroborating the segmented regression findings reported in Table 3(a) of the main text. The Chow test evaluates the joint significance of β_2 and β_3 together; this is why the Immunization Coverage series shows a highly significant Chow F-statistic despite non-significant individual β_2 and β_3 coefficients in Table 3(a) — the joint test has more statistical power than either individual coefficient test alone, particularly in a short series.

Supplementary Table S3: Sensitivity Analysis for Multicollinearity in the Exploratory MMR Regression Model

As described in Section 2.5 (Stage 4) of the main text, the full MMR regression model (Health Budget, Insurance Coverage, Outpatient Visits) exhibited substantial predictor intercollinearity. The Variance Inflation Factor (VIF) values reported in the main manuscript (Table 4b) – 82.24 for health budget, 17.58 for insurance coverage and 65.03 for outpatient visits- confirm severe multicollinearity among these conceptually related predictors this pattern is expected because the predictors represent interdependent components of an integrated health-system reform rather than interdependent components of an integrated health-system reform rather than independent policy variables. Consequently, the multivariable regression analyses should be interpreted as exploratory and hypothesis-generating and the individual regression coefficients should not be interpreted as independent causal effects (O'Brien, 2007). To assess whether this instability affects the study's substantive conclusions, three complementary specifications were compared: the full model, a reduced-predictor model

omitting the most collinear variable (Health Budget), and a ridge-regularized model with standardized predictors across a range of shrinkage penalties.

Table S3a. Full vs. Reduced-Predictor Model Comparison (MMR)

Term	Full Model	Reduced Model (Budget dropped)	VIF (reduced)
Intercept	54.81	143.86	—
Health Budget	-0.0143	—	—
Insurance Coverage	0.174	-0.552	13.90
Outpatient Visits	1.538*	-1.292	13.90
R^2	0.995	0.952	—

**Health Budget was removed from the reduced model to evaluate the robustness of coefficient estimates under reduced multicollinearity. Although variance inflation factors decreased, collinearity remained substantial. The reduced model retained a high coefficient of determination (R^2 = 0.952), supporting the interpretation that the observed associations primarily reflect a shared temporal trend among health-system variables rather than the independent contribution of individual predictors.*

Segmented regression model:

$$Y_t = \beta_0 + \beta_1(Time_t) + \beta_2(Intervention_t) + \beta_3(TimeAfterIntervention_t) + \varepsilon_t$$

Table S3b. Ridge Regression — Standardized Coefficients Across Shrinkage Penalties (MMR)

Predictor	α = 1	α = 5	α = 10
Health Budget	-9.22	-6.26	-5.39
Insurance Coverage	-4.06	-4.95	-4.69
Outpatient Visits	-4.64	-5.28	-4.89

Standardized (z-scored) predictors; coefficients represent the change in MMR (in standard-deviation units) per one-standard-deviation change in each predictor. Unlike the unstable, sign-inconsistent OLS coefficients under severe collinearity, all three predictors show a consistent negative association with MMR across all tested shrinkage penalties, supporting the interpretation offered in the main text: these variables jointly track a shared health-system-strengthening trend rather than exerting independent, separable effects, and no single predictor's apparent OLS coefficient (including its sign) should be over-interpreted.

Supplementary Figure S1: Residual Diagnostic Plots

Residual-versus-fitted and residual-versus-time plots for the three segmented regression (ITS) models reported in Table 3(a) of the main text. Residuals show no evident systematic patterning with respect to fitted values or time, and no marked change in residual variance before versus after the 2017 policy break, consistent with the autocorrelation and stationarity diagnostics reported in Table 2 of the main text.

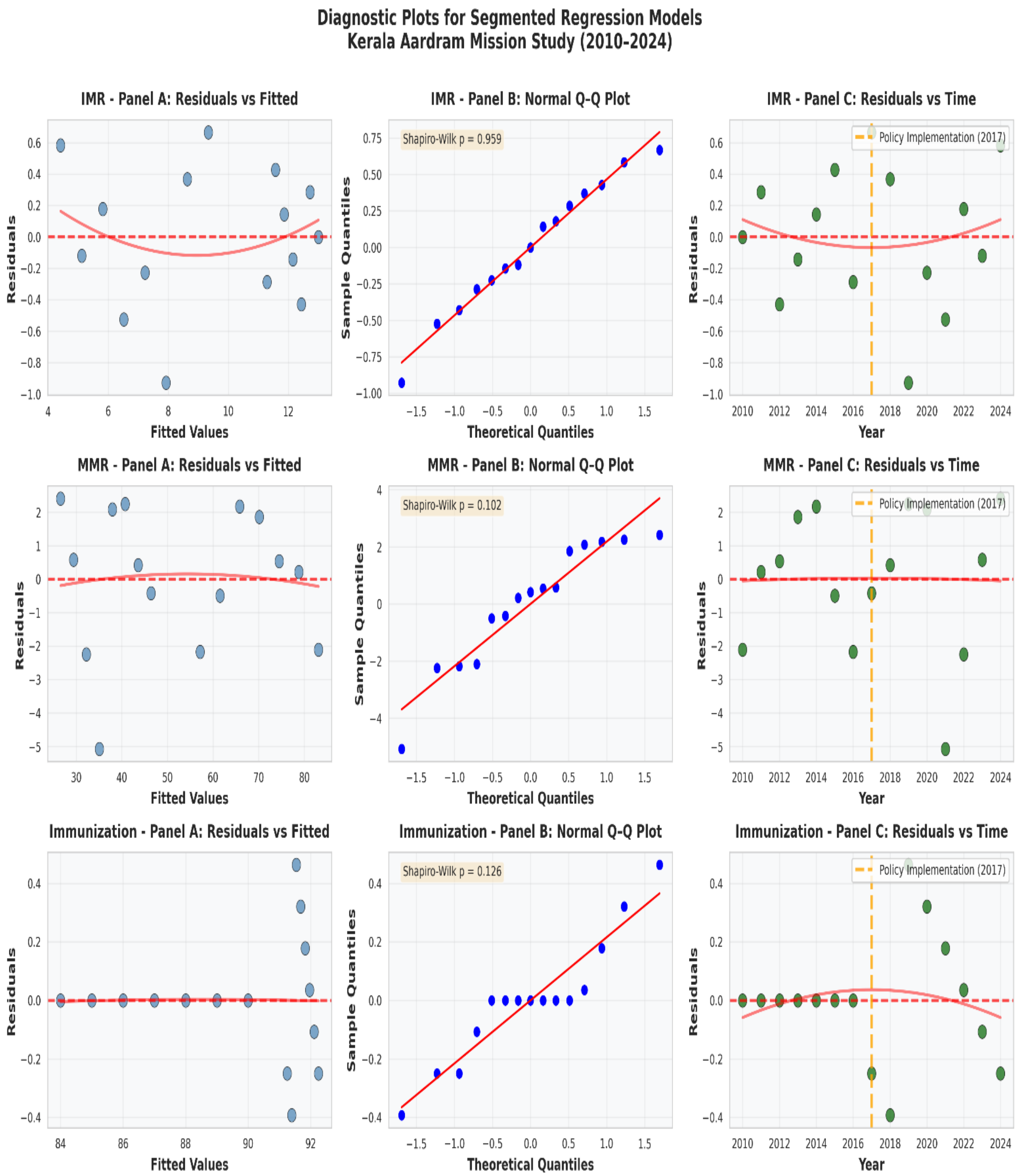

Nine-panel diagnostic figure showing residuals versus fitted values, normal Q–Q plots, and residuals versus time for segmented regression models of Infant Mortality Rate, Maternal Mortality Ratio, and Full Immunization Coverage in Kerala (2010–2024). A vertical dashed line marks the Aardram Mission implementation in 2017.

Data Availability Statement

The secondary administrative datasets used in this analysis are from publicly available government sources: Sample Registration System reports (https://censusindia.gov.in), Health at a Glance: Kerala annual reports (Government of Kerala Health Department), and Kerala State Budget Documents (Government of Kerala Finance Department). NFHS and NSS microdata are available from the Demographic and Health Surveys Program (https://dhsprogram.com) and the National Data Archive (India), respectively, subject to user registration and data-sharing agreements. All analytical code and the derived state-level dataset (aggregated annual time series 2010–2024) are available from the corresponding author upon reasonable request. No novel primary data were collected; all analyses are based on secondary sources as documented in Section 2.3 of the main text and Supplementary Table S1 above.

Government of Kerala – Health at a Glance Report: Government of Kerala, Directorate of Health Services. Health at a Glance 2023–24. Thiruvananthapuram: Directorate of Health Services, Government of Kerala, 2024. https://dhs.kerala.gov.in/wp-content/uploads/2025/05/H%40G-2023-24-1.pdf.

National Family Health Survey (NFHS): International Institute for Population Sciences (IIPS) and ICF. National Family Health Survey (NFHS-5), 2019–21: India Report. Mumbai: IIPS, 2021. https://www.nfhsiips.in.

Kerala Health Budget Report: Government of Kerala, Finance Department. Kerala Budget in Brief 2025–26. Thiruvananthapuram: Government of Kerala, 2025. https://budget.kerala.gov.in/keralabudgetdoc/2025_26/BIB.pdf.

National Sample Survey (NSS): Ministry of Statistics and Programme Implementation (MoSPI). Household Social Consumption on Health, NSS 75th Round (July 2017–June 2018). New Delhi: Government of India, 2019. https://mospi.gov.in.

Sample Registration Systems (SRS): Office of the Registrar General and Census Commissioner, India. Sample Registration System Statistical Report 2021. New Delhi: Government of India, 2023. https://censusindia.gov.in.

Demographic and Health Survey (DHS): The DHS Program. Demographic and Health Surveys Methodology and Datasets. Rockville, MD: ICF International. https://dhsprogram.com.

Supplementary References

Bernal JL, Cummins S, Gasparrini A. Interrupted time series regression for the evaluation of public health interventions: a tutorial. Int J Epidemiol (2017) 46:348–355. doi: 10.1093/ije/dyw098

Chow GC. Tests of equality between sets of coefficients in two linear regressions. Econometrica (1960) 28:591–605. doi: 10.2307/1910133

Jandoc R, Burden AM, Mamdani M, Levesque LE, Cadarette SM. Interrupted time series analysis in drug utilization research is increasing: systematic review and recommendations. J Clin Epidemiol (2015) 68:950–956. doi: 10.1016/j.jclinepi.2014.10.011

O'Brien RM. A Caution Regarding Rules of Thumb for Variance Inflation Factors. Qual Quant (2007) 41:673–690. doi: 10.1007/s11135-006-9018-6
